# The Tumor Microenvironment in the Response to Immune Checkpoint Blockade Therapies

**DOI:** 10.3389/fimmu.2020.00784

**Published:** 2020-05-07

**Authors:** Florent Petitprez, Maxime Meylan, Aurélien de Reyniès, Catherine Sautès-Fridman, Wolf H. Fridman

**Affiliations:** ^1^Programme Cartes d'Identité des Tumeurs, Ligue Nationale Contre le Cancer, Paris, France; ^2^Centre de Recherche des Cordeliers, INSERM, Sorbonne Université, Université de Paris, Equipe inflammation, complément et cancer, Paris, France

**Keywords:** tumor microenvironment, immunotherapy, immune checkpoint blockade, response, prediction

## Abstract

Tumor cells constantly interact with their microenvironment, which comprises a variety of immune cells together with endothelial cells and fibroblasts. The composition of the tumor microenvironment (TME) has been shown to influence response to immune checkpoint blockade (ICB). ICB takes advantage of immune cell infiltration in the tumor to reinvigorate an efficacious antitumoral immune response. In addition to tumor cell intrinsic biomarkers, increasing data pinpoint the importance of the TME in guiding patient selection and combination therapies. Here, we review recent efforts in determining how various components of the TME can influence response and resistance to ICB. Although a large body of evidence points to the extent and functional orientation of the T cell infiltrate as important in therapy response, recent studies also confirm a role for other components of the TME, such as B cells, myeloid lineage cells, cancer-associated fibroblasts, and vasculature. If the ultimate goal of curative cancer therapies is to induce a long-term memory T cell response, the other components of the TME may positively or negatively modulate the induction of efficient antitumor immunity. The emergence of novel high-throughput methods for analyzing the TME, including transcriptomics, has allowed tremendous developments in the field, with the expansion of patient cohorts, and the identification of TME-based markers of therapy response. Together, these studies open the possibility of including TME-based markers for selecting patients that are likely to respond to specific therapies, and pave the way to personalized medicine in oncology.

## Introduction

Cancers arise from the accumulation of genomic abnormalities in pre-malignant cells. These cells hijack key homeostasis functions to promote their survival and growth and avoid elimination by the immune system (1). The interplay between malignant cells and the immune system during cancer development has been proposed to comprise three steps: elimination, followed by an equilibrium phase, and escape from the immune control, termed the 3 Es of cancer immunoediting (2).

Indeed, malignant cells develop and evolve in a complex and strongly interconnected tumor microenvironment (TME), comprising a vast variety of immune cells and non-immune stromal cells such as endothelial cells and fibroblasts (3). Studying the TME is of paramount importance given the clinical impact of its composition and extent (4). For instance, a strong infiltration by CD8^+^ T cells is generally associated with a favorable prognosis (5–8), while the presence of M2-polarized macrophages is widely considered a negative prognostic marker (9–11). Moreover, the TME, through its many components, harbors a high diversity of possible targets for cancer treatment (4, 12, 13).

In recent years, therapeutic options for the treatment of cancer have changed tremendously with the development of immunotherapy. Among the various types of immunotherapy, immune checkpoint blockade (ICB) covers a range of monoclonal antibody-based therapies that aim at blocking the interaction of inhibitory receptors (immune checkpoints) expressed on the surface of immune cells, with their ligands. The main targets for these treatments are CTLA-4 and PD-1 or its ligand PD-L1. ICB has drawn considerable attention (14, 15), especially because of the durability of responses and effects on patients' overall survival. A key challenge is identifying patients who are the most likely to respond.

Several markers have recently been suggested to be associated with response to ICB. The PD-1/PD-L1 axis is at the forefront of interactions between immune, stromal and tumor cells. The expression of both PD-1 and PD-L1 was shown to be increased in melanoma patients who responded to PD-1 blockade (16). PD-L1 expression on tumor cells was associated with response to anti-PD-1 therapies in various malignancies (17, 18). To date, PD-L1 detection by immunohistochemical analysis is the only companion test approved by the FDA for ICB in NSCLC, urothelial carcinoma, cervical cancer, and triple-negative breast cancer (19). However, subsequent trials have reported conflicting results for the use of PD-L1 as a predictive biomarker (20), likely due to the heterogeneity of modalities used (such as the antibodies used for detection, or the PD-L1 positivity threshold). In addition, it was shown, initially in melanoma and non-small cell lung cancer (NSCLC) which are highly mutated tumor types (21), that the higher the mutational burden of a tumor, the more likely it is to respond to ICB (22–24). This was recently demonstrated to remain true in many malignancies (25). In particular, a high response rate to ICB was reported in tumors with mismatch-repair deficiency (26–28). However, this is only a general correlate that does not provide sufficient sensitivity or specificity in all cancer types (29). Recently, the gut microbiome was also shown to be associated with response to ICB (30–33), although many questions remain open in this area (34).

**Figure 1:**
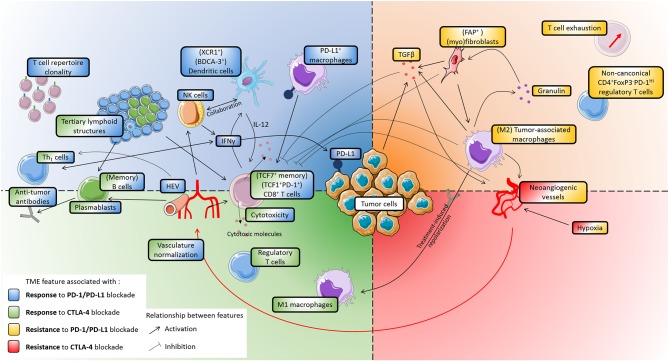
Main features of the tumor microenvironment that influence patients' response to immune checkpoint blockade. The figure is divided in four quarters, corresponding to association with either response or resistance, to either CTLA-4 or PD-1/PD-L1 blockade. Activating or inhibitory relationships between TME features are indicated by black arrows. Whenever phenotypes are indicated between brackets, it indicates that studies identified particular subsets as being associated with patients' response. PD-L1 is the only target of ICB that is shown, as it is the only one which expression was directly shown to associate with patients' response. **Upper left** (blue), features associated with increased response to PD-1/PD-L1 blockade: T cell repertoire clonality, NK cells, Dendritic cells, PD-L1+ macrophages, high endothelial venules, IFNγ. **Lower left** (green), features associated with increased response to CTLA-4 blockade: M1-polarized macrophages, regulatory T cells. **(Left)** (green and blue), some features are associated with an increased response to both CTLA-4 and PD-1/PD-L1 bloackade: CD8+ T cells, cytotoxicity, tertiary lymphoid structures, Th1 cells, B cells, plasmablasts, antibodies, vasculature normalization. **Upper right** (yellow), features associated with resistance to PD-1/PD-L1 blockade: M2-polarized macrophages, granulin, fibroblasts, TGFβ, T cell exhaustion, non-canonical regulatory T cells. **Lower right** (red): no markers were identified as being associated solely with resistance to CTLA-4 blockade. **(Right)** (yellow and red), features associated with resistance to both CTLA-4 and PD-1/PD-L1 blockade: hypoxia, neoangiogenic vessels. Cell drawings originate from Servier Medical Art (https://smart.servier.com), distributed under a CC-BY 3.0 Attribution license (https://creativecommons.org/licenses/by/3.0/).

Here, we review recent advances in understanding the composition and functionality of the TME in response and resistance to ICB, and we discuss how these insights can facilitate the prediction of patient responses. The association of TME components with response to ICB is summarized in Table 1 (factors associated with response) and Table 2 (factors associated with resistance), as well as in Figure 1.

**Table 1:** Summary of TME components associated with response to immune checkpoint blockade.

**Microenvironment marker**	**Checkpoint blockade**	**Cancer types**	**References**
**T CELL MARKERS**			
CD8^+^ T cell density	PD-1	Melanoma	(16)
	PD-L1	Multiple malignancies	(35)
Augmentation of cytotoxicity	PD-1	Melanoma	(36)
	CTLA-4	Melanoma	(37)
Memory-like CD8^+^TCF7^+^ T cells	PD-1	Melanoma	(38)
Tcf1^+^PD-1^+^CD8^+^ T cells	PD-1/CTLA-4	Melanoma mouse model	(39)
CD4^+^ Th1 cells	CTLA-4	Melanoma	(40)
	PD-1, CTLA-4	Sarcoma mouse model	(41)
FoxP3^+^ regulatory T cells	CTLA-4	Melanoma	(42)
T cell repertoire clonality	PD-1	Melanoma	(16, 36, 43, 44)
	PD-L1	Multiple malignancies	(45)
IFNγ	PD-1, PD-L1	Multiple malignancies	(46–49)
**B CELLS AND TERTIARY LYMPHOID STRUCTURES**			
B cells	PD-1	Soft-tissue sarcoma	(50)
	PD-1	Melanoma	(38)
	PD-1, CTLA-4	Melanoma	(51, 52)
	PD-1, CTLA-4	Breast cancer mouse model	(53)
	PD-1, PD-L1, CTLA-4	Melanoma, urothelial carcinoma	(54)
Memory B cells	PD-1	Melanoma	(55)
Plasmablasts	PD-1	Melanoma	(55)
	PD-1, CTLA-4	Melanoma, lung cancer, renal cell carcinoma	(56)
Tertiary lymphoid structures	PD-1	Soft-tissue sarcoma	(50)
	PD-1	Pancreatic cancer mouse model	(57)
	PD-1, CTLA-4	Melanoma	(51, 52)
Antibodies	PD-1, CTLA-4	Melanoma, clear cell renal cell carcinoma	(58)
	PD-1	HPV-related cancers	(59)
**INNATE IMMUNE CELLS**			
Dendritic cells	PD-1	Colorectal and melanoma mouse models	(60)
	PD-L1	Renal cell carcinoma, NSCLC	(61)
XCR1^+^ dendritic cells	PD-L1	Renal cell carcinoma	(62)
BDCA-3^+^ dendritic cells	PD-1	Melanoma	(63)
PD-L1^+^ macrophages	PD-1, PD-L1	NSCLC	(64)
M1 macrophages	CTLA-4	Melanoma	(65)
NK cells	PD-1	Melanoma	(63)
	PD-1, PD-L1	Mouse models	(66)
**STROMAL TME MARKERS**			
Tumor vasculature normalization	PD-1, CTLA-4	Mouse models	(67–69)
High endothelial venules	PD-1, PD-L1	Mouse models	(70)

**Table 2:** Summary of TME components associated with resistance to immune checkpoint blockade.

**Microenvironment marker**	**Checkpoint blockade**	**Cancer types**	**References**
**T cell markers**			
Exhausted T cells	PD-1	Lung cancer (human and mouse models)	(71, 72)
Non canonical CD4^+^FoxP3^−^ regulatory T cells	PD-1	Melanoma mouse models	(73)
Follicular helper T cells	CTLA-4	Melanoma mouse models	(73)
**Innate immune cells**			
Macrophages	PD-1	Lung squamous cell carcinoma, pancreatic ductal adenocarcinoma	(74, 75)
**Stromal TME markers**			
Hypoxia	PD-1, CTLA-4	Melanoma and prostate cancer mouse models	(76, 77)
TGFβ signaling	PD-L1	Urothelial cancer, colorectal cancer mouse model	(78, 79)

## Importance of the TME in Response and Resistance to Immune Checkpoint Blockade Therapy

### T Cells Generally Favor ICB Efficacy but Their Diversity and Functionality Are Crucial

ICB therapies were designed to reinvigorate efficacious anti-tumor immune responses mainly mediated by T cells (80, 81). The relationship between the density and localization of CD8^+^ T cells and the response to PD-1 blockade in melanoma (16), as well as to PD-L1 blockade in various cancer types (35), has been analyzed, and a higher density of CD8^+^ T cells, both in the tumor core and in the invasive margin, was shown to correlate with an increased response to PD-1/PD-L1 blockade. A metric known as the Immunoscore (5), that accounts for the density of CD3^+^ and CD8^+^ T cells in the tumor core and the invasive margin and has prognostic value (6), could be used to predict patient response to ICB (82, 83).

However, the mere presence of CD8^+^ T cells is not entirely informative of whether patients are likely to respond to ICB. The phenotype and functionality of these cells also need to be analyzed. The development of single-cell RNA sequencing technology has highlighted the high variability that is inherent to tumor-infiltrating T cells in many malignancies (38, 84–91). In melanoma, dysfunctional CD8^+^ T cells were shown to form a major proliferative compartment (91). Interestingly, in mouse models, some dysfunctional CD8^+^ T cells are nonetheless tumor-specific (92) and a subpopulation can be reactivated (92, 93). Hence, the cytotoxicity of CD8^+^ T cells must be considered. A study investigating the changes in the TME during anti-PD-1 treatment of anti-CTLA-4-resistant melanoma patients reported that, although the density of tumor-infiltrating lymphocytes did not significantly change, their cytolytic activity increased (36). Pre-treatment expression of cytotoxicity-related genes, notably *GZMA*, is also related to PD-1 blockade response (43). In melanoma, a higher expression of gene signatures related to T cell cytotoxicity was associated with the clinical activity of the anti-CTLA-4 ipilimumab (37). Moreover, regardless of the overall T cell infiltration, an increased presence of memory-like CD8^+^TCF7^+^ T cells (as opposed to generally exhausted CD8^+^TCF7^−^ T cells) is associated with an increased response to PD-1 blockade in patients with metastatic melanoma (38).

The TME harbors both tissue-resident T cells and newly infiltrating T cells that enter via the bloodstream. The involvement of tissue-resident T cells in ICB has been investigated. In lung cancer, tumors with a higher expression of tissue-resident memory cell marker CD103 on tumor-infiltrating cytotoxic lymphocytes displayed an enhanced T cell cytotoxicity, independently of an increased cytotoxic T cell density (94). In melanoma, a subset of T cells expressing TCF1 [a marker of self-renewable CD8^+^ T cells (54, 95) that proliferate after PD-1 blockade (54)] and PD-1 was identified (39). In mice, these Tcf1^+^PD-1^+^ tumor infiltrating lymphocytes were shown to be able to proliferate, and to mediate durable responses to ICB (39). Thus, rather than rejuvenating differentiated CD8^+^ T cells, ICB seems to act on less-differentiated memory-like CD8^+^ T cells that expand into a pool of effector CD8^+^ T cells.

CD4^+^ T cell subsets also play critical roles in tumor immunology and immunotherapy (96). Several subsets of effector CD4+ Th_1_ cells have been shown to be more abundant in melanoma tumors responding to CTLA-4 inhibition (40). Moreover, a recent study in a sarcoma mouse model revealed that presentation of peptides by tumor cells on class II major histocompatibility complex (MHC) and their recognition by CD4^+^ Th_1_ cells was crucial to generate functional CD8^+^ T cell responses during ICB with anti-PD-1, anti-CTLA-4, or combination of both (41).

CD4^+^FoxP3^+^ regulatory T cells (Treg) are generally associated with a poor prognosis in several cancers as suppressors of antitumor immune responses (97). However, since CTLA-4 is constitutively expressed on Tregs, their presence has been shown to be associated with response to CTLA-4 blockade (42). Interestingly, another study (98) revealed that the efficacy of CTLA-4 inhibition relied on Treg depletion during treatment. The impact of anti-CTLA-4 treatment on the relative composition of the T cell infiltrate is disputed. An increase in CD4^+^ and CD8^+^ T cell infiltration is observed, and in one study, the Treg compartment was not depleted (99), which was not the case in other works (98). Tregs have also been related to hyperprogression, i.e., ICB-related tumor growth acceleration. For instance, in gastric cancer, it was shown that PD-1 blockade increased Treg infiltration, which subsequently promoted hyperprogression (100). Non-conventional inhibitory T cells have also been studied with regards to ICB (73). These CD4^+^FoxP3^−^PD-1^Hi^ (4PD1^hi^) T cells, which have immunosuppressive functions and present similarities with follicular helper T cells, have distinct behaviors in tumors treated by PD-1 or CTLA-4 blockade. CTLA-4 inhibition increases the number of 4PD1^hi^ T cells while PD-1 blockade depletes them and mitigates their inhibitory functions. The persistence of these cells after PD-1 blockade is considered a negative prognostic factor (100).

To mediate a potential antitumor response, T cells must be tumor-reactive, i.e., recognize a peptide presented by the MHC on tumor cells. Several parts of this mechanism are closely related to ICB response (101). Some studies reported that T cell receptor repertoire clonality, i.e., the expansion of specific T cell clones, was associated with response to PD-1 blockade (16, 36, 43, 44). Similar results were reported for PD-L1 blockade in several lung, endometrial, colorectal and kidney malignancies, and this clonality was also observed in peripheral T cells, which may open the way for non-invasive testing (45). Downregulation of antigen presenting machinery and loss of heterozygosity of class I HLA genes also impair response to ICB (102–104). In melanoma, class II MHC profiling has been proposed as a biomarker of response to ICB, alone (105, 106) or in combination with PD-L1 detection (107).

The central position of T cells in immunotherapy is also highlighted by the pivotal role of interferon gamma (IFNγ) in the interplay between TME components and in mediating response to ICB. IFNγ can be secreted by many different cell types, notably cytotoxic T cells, Th_1_ and NK cells. It promotes cytotoxicity of CD8^+^ T cells and NK cells, favors Th_1_ polarization of CD4^+^ T cells, upregulates antigen presentation and activates M1 macrophages. Furthermore, it inhibits cell growth and favors apoptosis (108). IFNγ also upregulates PD-L1 expression on neighboring cells (109). IFNγ signaling was shown to be associated with a higher response rate to PD-(L)1 blockade in NSCLC (48, 49), urothelial carcinoma (48) and melanoma (46, 49). A T-cell inflamed gene expression signature strongly correlating with IFNγ signaling proved efficient in predicting response to PD-1 blockade in a pan-cancer study (47), suggesting this may have broader significance.

### B Cells and Tertiary Lymphoid Structures: Emerging Factors Associated With Increased ICB Response

Although T cells are key effectors of antitumor immunity, there is a growing interest in the role of B cells for ICB. Although there have been reports that B cell absence or depletion did not prevent anti-PD-1 efficacy in melanoma (110), other studies have indicated a favorable impact of B cells on ICB. Single-cell studies of tumor-infiltrating immune cells revealed that melanoma tumors responding to PD-1 blockade exhibited increased numbers of B cells (38). A recent re-analysis of the same dataset revealed that subsets of B cells varied strongly between responders and non-responders, with higher infiltration by memory-like B cells and plasmablast-like cells in tumors of responders (55). An increased presence of plasmablasts had previously been described in the blood of melanoma, lung, and renal cell carcinomas patients responding to anti-CTLA-4 and anti-PD-1 therapies (56). A gene signature related to memory B cells (111) was reported to be associated with clinical benefit and improved survival in anti-PD-1- and anti-CTLA-4-treated melanoma patients and in anti-PD-L1-treated urothelial carcinoma (112). B-cell-secreted antibodies have also been related to ICB response. In particular, higher quantities of melanoma-specific antibodies were found in the serum of patients responding to CTLA-4 or PD-1 inhibition (58). In HPV-related cancers treated by PD-1 blockade, increased IgG and IgM antibody response was found in the serum of responders but not non-responders (59). In clear cell renal cell carcinoma, IgG-containing immune complexes could lead to classical complement pathway activation, which was associated with a higher expression of immune checkpoint molecules (113). This suggests a relationship between B cells, complement system activation and immune exhaustion to be further explored in light of ICB advances. Moreover, since B cells can also express immune checkpoint molecules such as PD-1, principally on regulatory B cells (114), or CTLA-4 (115), it is likely that ICB treatment can also have a direct effect on B cells. Murine models of HPV-induced head and neck squamous cell carcinoma showed that radiotherapy and PD-1 blockade induced memory B-cells, plasma cells, and antigen-specific B-cells, as well B-cell germinal center formation (59).

B cells are a crucial component of tertiary lymphoid structures (TLS), lymph-node-like lymphocytic aggregates that are sites for activation and maturation of antitumor immune responses (116). Recent studies addressed the question of the impact of tumor infiltration by B cells and presence of TLS on patient survival and response to PD-1 blockade in soft-tissue sarcoma (50) and to PD-1 and CTLA-4 blockade in melanoma (51, 52). These studies reveal that groups of patients marked by the presence of TLS and a stronger infiltration by B cells present a higher response rate to ICB. Work on murine models of breast cancer suggest an activation of B cells and follicular helper T cells (Tfh), a T cell subset found in TLS, by ICB, and an influence of these cells on response (53). Moreover, induction of TLS improved the efficacy of ICB in checkpoint-blockade-resistant tumors in a mouse model (57). Presence of CXCL13-producing PD-1^Hi^ CD8^+^ T cells in TLS has been associated with response to PD-1 blockade in NSCLC (117). However, mice deficient for Tfh were shown to present an improved response to CTLA-4 blockade (73). Therefore, the impact of TLS on ICB response is likely to have a broad applicability, although not universal.

### Innate Immune Cells: Functionality Is the Key

Besides the adaptive immune cells presented above, the immune TME can also harbor various innate immune populations, such as dendritic cells, macrophages, or NK cells, with critical effects on tumor evolution. Increasing efforts focus on exploiting them to treat cancer (118). Moreover, functionality-based subtypes of each of these populations have been shown to influence response or resistance to ICB.

To exert effective antitumor activity, CD8^+^ T cells require priming by antigen presenting cells, such as dendritic cells (DC). Dendritic cells can be classified as classical dendritic cells (cDCs) and specialized type I interferon-producing plasmacytoid dendritic cells (pDCs). cDCs are further divided into cDC1 with enhanced exogenous antigen cross-presentation to CD8^+^ T cells, and cDC2 cells which favor presentation to CD4^+^ T cells inducing Th-2 or Th-17 responses (96, 119). A recent study using mouse models revealed that effective PD-1 blockade therapy requires the presence of IL-12-producing cDC1 (60). During anti-PD-1 treatment, DCs respond to IFNγ produced by neighboring T cells by secreting IL-12, thereby enhancing T-cell-mediated tumor cell killing (60). In renal cell carcinoma, a study reported that a higher expression of genes related to cross-presenting DCs, including *XCR1*, was associated with better survival of patients treated with PD-L1 blockade (62). A stimulatory DC subset, identifiable by expression of BDCA-3 and CLEC9A markers (120), is associated with anti-PD-1 immunotherapy response in melanoma patients (63). The BDCA-3^+^CLEC9A^+^ DC subset (which produces high levels of IFNα) induces Th1 polarization and exhibits superior capacity to cross-present dead cell-associated antigens (121), suggesting that it plays an essential role in the induction of antitumor responses. Interestingly, this subset also expresses chemokine receptor XCR1 and interacts with XCL1-producing NK cells, which were also found to be associated with anti-PD-1 response in melanoma (63). PD-L1 can also bind CD80, a co-stimulatory molecule expressed on DCs, and repress its availability (61). It was shown that PD-L1 inhibition could allow a higher expression of CD80 on DCs and therefore a stronger priming of T cell responses through the binding of CD80 with CD28 on T cells. In the same study, a DC gene signature (including *XCR1, BATF3, FLT3*, and *IRF8*) expression correlated with increased survival of patients treated with PD-L1 blockade in NSCLC and renal cell carcinoma (61).

Macrophages form another critical innate immune population, capable of phagocytosis, and antigen presentation. Although their functionality spectrum can be more complex, two opposite states of polarization are often distinguished. Classically activated M1 macrophages favor CD8^+^ T cell activation through antigen presentation and cytokine secretion. In contrast, alternatively activated M2 macrophages have pro-angiogenic and immunosuppressive properties and secrete TGFβ (122). In NSCLC, the expression of PD-L1 on immune cells is mostly found on the surface of CD68^+^ macrophages. This macrophage expression of PD-L1 is associated with an increased CD8^+^ T cell infiltration in tumors and an increased survival of patients when they are treated with PD-(L)1 blockade therapies (64). In lung squamous cell carcinoma, stromal macrophages, activated through CSF-1R (the receptor of colony-stimulating factor-1 (CSF-1), a macrophage growth factor), trap CD8^+^ T cells and prevent them from entering the tumor core, thereby limiting the efficacy of PD-1 blockade therapy (74). This phenomenon was also reported in liver metastases of pancreatic ductal adenocarcinoma, in which it was shown that CSF-1 induced granulin expression by macrophages. This led to the recruitment of myofibroblasts that retained CD8^+^ T cells outside of the tumor (75). Tumor-associated macrophages (TAMs) can also have other immunosuppressive effects (123), through secretion of IL-10 and TGFβ and the expression of immune checkpoint molecules PD-L1, PD-L2, CTLA-4 ligands CD80 and CD86 (124), and PD-1 (125). M2-polarized macrophages could also contribute to hyperprogression. Indeed, a study in NSCLC showed that patients with hyperprogression under PD-(L)1 inhibition showed increased tumor infiltration by M2 macrophages, and suggested that macrophage reprogramming to the M2 phenotype might have occurred as a result of the binding of treatment antibodies to macrophage Fc receptors (126). Interestingly, PD-1 can be expressed on macrophages that are mostly of the protumor M2 phenotype, and PD-(L)1-blockade therapy could revert their function toward an antitumor M1 phenotype that can engage in malignant cell phagocytosis (125). Moreover, in melanoma patients, a higher infiltration by CD68^+^CD16^+^ classically activated (M1) macrophages was found in tumors of responders to CTLA-4 as compared to non-responders (65).

NK cells are innate lymphocytes that do not have antigen-specific receptors. They are able to sense cells lacking expression of class I MHC and can exert cytotoxic functions or secrete cytokines. Evidence point to the presence of PD-1^+^ NK cells with an activated phenotype (127, 128). However, the binding with PD-L1 mediates a dysfunction of these NK cells (127, 129). Consequently, NK cells are likely to weigh in PD-1-axis inhibition. This is reinforced by the fact that, in mouse models, depletion of NK cells abolished the effects of PD-1 and PD-L1 inhibition (66). Notably, several novel checkpoint inhibitors targeting NK cells are being tested in clinical trials (118, 127).

### Evolution of the TME During Treatment and Response

The composition of the TME is dynamic and evolves during ICB therapies. Several studies have pointed to differential evolution of the TME between responders and non-responders. In longitudinal studies of melanoma patients treated with ICB (36, 130, 131). Interestingly, the TME composition differences between responders and non-responders were found to be stronger early on-treatment than before ICB (130, 131). Indeed, the differences in the densities of CD4^+^ or CD8^+^ T cells were more profoundly visible after two or three anti-PD-1 doses than at baseline (130). Similarly, analysis of on-treatment biopsies revealed a difference between responders and non-responders in the expression of PD-1 (130) or PD-L1 (130, 131). These observations stand for either anti-CTLA-4-progressors treated with PD-1 blockade (130), or for patients treated with PD-1 inhibitor alone or in combination with CTLA-4 blockade (131). Response to ICB is accompanied by more global changes in the TME composition. An increase in the numbers of CD8+ T cells and NK cells in tumors of responders to PD-1 inhibitors, as well as a decrease in macrophage infiltration were shown (36). Tumors responding to ICB also revealed an increase in B cells and TLS (51, 52). T cell receptor (TCR) repertoire clonality of responders was found to be increased during therapy (36, 131). In a trial combining CTLA-4 blockade with an demethylating agent, an increase in CD8+ T cell and PD-1 expression was reported (132).

The functionality of the TME can also be significantly impacted by ICB. During PD-1 blockade, responders showed an increased expression of the T-cell exhaustion marker LAG3 (130). Conversely, the upregulation of exhausted T cells during PD-1 therapy, as seen by expression of alternative inhibitory checkpoints LAG-3 or TIM-3, has been linked to adaptive resistance to PD-1 treatment in lung cancer in humans (71) and in mouse models (72). In NSCLC and melanoma patients, a higher expression of CD38 in patients with high basal or treatment-induced T cell infiltration was associated with adaptive resistance to PD-(L)1 inhibition (133). Interestingly, PD-1 blockade was also found to induce clonal replacement preferentially of exhausted CD8+ T cells compared to any other T cell subset (134). This could mean that T cells present at baseline may show reduced proliferation, and that the response to ICB could be due to T cell clones that enter the tumor during the course of treatment.

### Stromal Tumor Microenvironment: Complex Interactions With Immune and Tumor Cells

The TME forms a complex system in which tumor cells interact not only with immune cells but also with non-immune stromal cells. The two main stromal cell types are endothelial cells, forming vessels, and fibroblasts. Since blood and lymphatic vessels partly govern the extent of the immune infiltration of the tumor, their involvement in response to ICB has been analyzed. In hypoxic conditions, tumors activate neoangiogenesis, forming abnormal vasculature that has been proposed to reduce the infiltration of lymphocytes (135). This neoangiogenic vasculature can be specifically targeted to normalize the vasculature. Several studies have demonstrated that normalizing the vasculature facilitated the entry of immune cells into the tumor, and may therefore enhance responsiveness to ICB (67, 69). This approach is further supported by in breast and colon murine models treated with PD-1 or CTLA-4 blockade, response to ICB was associated with increased vessel perfusion, which is a measure of proper vascular function (68). This could explain the success of combinatory treatments with anti-angiogenic drugs and PD-1 or PD-L1 blockade in mouse tumor models (70, 136), which relies on the normalization of the tumor vasculature (136) and promotion of high endothelial venules (70), a vessel type specialized in lymphocyte trafficking and found in secondary lymphoid organs and TLS (137). Hypoxia, which leads to neoangiogenic vessel formation, is also implicated in response to ICB. In murine models of melanoma (76) and prostate cancer (77), targeting hypoxia products led to the sensitization of the tumors to ICB.

Fibroblasts, which are the other main stromal population in the TME, can also play a critical role in response or resistance to ICB. Fibroblasts expressing the marker fibroblast activation protein-alpha (FAP) induced resistance to PD-1 inhibitors in colorectal cancer murine models by promoting immunosuppression, recruiting myeloid cells and inhibiting T cell activity (138). In metastatic pancreatic cancer, a decrease in αSMA^+^ fibroblast numbers by targeting macrophages has been associated with enhanced response to PD-1 blockade (75). Two recent studies point to the role of TGFβ in stroma-mediated resistance to PD-L1 blockade (78, 79). In a murine model of colorectal cancer with TGFβ-activated stroma, blockade of PD-L1 only leveraged limited responses, but resistance was overcome upon combination with TGFβ inhibition (78). Similar findings were observed in a model of metastatic liver cancer (78). This ICB-potentiating effect of TGFβ inhibition was also demonstrated in metastatic urothelial cancer patients (79). Increased TGFβ signaling was found in “immune-low” tumors, and TGFβ blockade co-administered with anti-PD-L1 showed anti-tumor strong responses in a murine urothelial cancer model, whereas neither treatment showed activity alone (79). Together, these results reveal the central role of stroma-derived TGFβ in inducing immune evasion and resistance to ICB.

## Conclusions

Immune checkpoint blockade has promoted a revolution of cancer treatment. Its mechanism of action is tightly related to the action of cytotoxic CD8^+^ T cells. However, a growing body of evidence points to relationships between patients' response and other components of the tumor microenvironment. The TME is indeed an intricated assembly of many cell populations that influence the abundance and functionality of the neighboring cells. Each cell population found in the TME, immune or stromal, influences response to ICB. This is particularly striking with the role of tertiary lymphoid structures, which combine T cells, B cells, and dendritic cells to allow a maturation and activation of an antitumoral immune response.

In the past years, several promising immune checkpoints that could also be inhibited have emerged. These notably include T cell-related inhibitory molecules such as Tim-3 or Lag-3, and TIGIT, expressed both by T cells and NK cells in the TME (139). However, there is still a lack or robust validated markers associated with response or resistance to these emerging treatments.

The future of research for biomarkers of response may be to analyze the TME composition as a whole instead of analyzing each population separately. To do so, many different approaches are available (140), including many computational approaches harnessing tumor transcriptomics (141), and are now extended to single-cell analyses. These investigations will be complemented by further studies of the locations of cells in the TME, by high-throughput multispectral imaging or spatial transcriptomics (142–144). The integration of these different approaches should allow the emergence of novel composite biomarkers that are necessary to predict patients' responses to the combination of antitumor and anti-TME therapies in the future.

## Author Contributions

FP, MM, and WF wrote the manuscript, which was amended and validated by all authors.

## Conflict of Interest

The authors declare that the research was conducted in the absence of any commercial or financial relationships that could be construed as a potential conflict of interest.
